# Sexually dimorphic effects of dietary sugar on lifespan, feeding and starvation resistance in *Drosophila*

**DOI:** 10.18632/aging.101335

**Published:** 2017-12-04

**Authors:** Bhakti Chandegra, Jocelyn Lok Yee Tang, Haoyu Chi, Nazif Alic

**Affiliations:** ^1^ Institute of Healthy Ageing and Department of Genetics, Evolution and Environment, University College London, WC1E 6BT, London, UK

**Keywords:** sexual dimorphism, ageing, Drosophila, diet

## Abstract

Lifespan and health in older age are strongly influenced by diet. Feeding *Drosophila melanogaster* diets high in sugar has increasingly been used as an experimental model to understand the physiological effects of unhealthy, contemporary human diets. Several metabolic parameters and physiological responses to nutrition are known to be dependent on the sex of the animal. However, sexual dimorphism in the responses to high-sugar diets in fruit flies has not been examined. Here we show that a high-sugar diet in *Drosophila melanogaster* elicits sexually dimorphic effects on feeding behaviour, starvation resistance and lifespan. Females feed less on such diets, while males feed more, and these feeding responses may have secondary consequences. Females, more than males, gain the ability to resist periods of starvation from high-sugar diets, indicating that the female response to excess sugar may be geared towards surviving food shortages in early life. At the same time, female lifespan is more susceptible to the detrimental effects of high sugar diets. Our study reveals differences between *Drosophila* sexes in their responses to sugar-rich diets, indicating the fruit fly could be used as a model to understand the sexually dimorphic features of human metabolic health.

## INTRODUCTION

There is an increasing proportion of older individuals in our societies [1]. Since age is the main risk factor for many chronic diseases [2], this demographic change is incurring rising personal and societal costs that need to be addressed.

Sex differences in longevity and age-old health are prevalent in the animal kingdom [3, 4]. In humans, females are longer lived but often bear a greater burden of age-related disease [4]. Similarly, in other species, females tend to be the longer-lived sex [3]. However, in many cases, this sexual dimorphism in lifespan is highly dependent on the environment [3, 4], indicating an important interaction between environmental conditions and sex. One such condition is the diet.

Diet has a profound effect on animal physiology, where relative and absolute levels of dietary components strongly impact reproductive success, health and adult lifespan [5, 6]. Indeed, recent changes in human diets are thought to contribute to the current pandemic of metabolic syndrome [5]. Similar to lifespan, metabolism and metabolic health in humans and other animals show sexually dimorphic characteristics [7–9], highlighting the importance of understanding sex differences in how diets shape animal health and longevity.

*Drosophila melanogaster* has often been used as a model to elucidate how diets impact longevity [10, 11]. In laboratory experiments, flies are often fed a diet of yeast, as a source of protein, and sucrose, as the main carbohydrate [10, 12]. Altering the absolute or relative amounts of these two dietary components has revealed that female lifespan is responsive to both and maximised at intermediate concentrations of each macronutrient [13, 14].

The impact of the relative amount of yeast (or protein) on lifespan is known to be sexually dimorphic in *Drosophila* [15]. Restricting the amount of yeast limits the female's reproductive output but increases her lifespan. However, this benefit of reducing dietary yeast is blunted in males. Recent work has shown that sex-specific differences in gut physiology [16] can account for this sexual dimorphism [15].

At the same time, excessive dietary sucrose has been used as a basis for a fly model of an unhealthy diet that may bare relevance to contemporary human diets. In flies, high-sugar diets have been shown to reduce lifespan and induce phenotypes akin to obesity, insulin-resistance, cardiomyopathy, diabetic nephropathy [14, 17–20], to interact with models of tumorigenesis [21] and display physiological effects persisting through life and across generations [22–24]. However, it remains unclear if the response of lifespan and other physiological traits to excessive sucrose is sexually dimorphic. One previous study, using a limited range of diets, detected a sexual dimorphism in lifespan [25], while another revealed that the sexes have different dietary optima for reproduction but did not detect a sexual dimorphism in the response of lifespan to a substantial increase in dietary carbohydrates [26].

Here we re-examine the effect of excessive dietary sugar on the lifespan in the two sexes, using an experimental paradigm optimized for ageing studies. We find that the female lifespan is more strongly shortened by sugar-rich diets. This sexually dimorphic response to excessive sugar is paralleled by sex differences in the modulation of feeding behaviour in response to the diet, which may account for the net reduction in female fecundity in the presence of excess sugar. Interestingly, we find that the effect of high-sugar diets on lifespan is inverted for starvation resistance, where such diets provide a benefit that is more evident in females than in males. This suggests that a female fly's handling of diets high in sugar is geared towards resisting periods of starvation at the expense of lifespan.

## RESULTS

### Dietary sugar content causes sexually dimorphic effects on *Drosophila* lifespan

**Figure 1:**
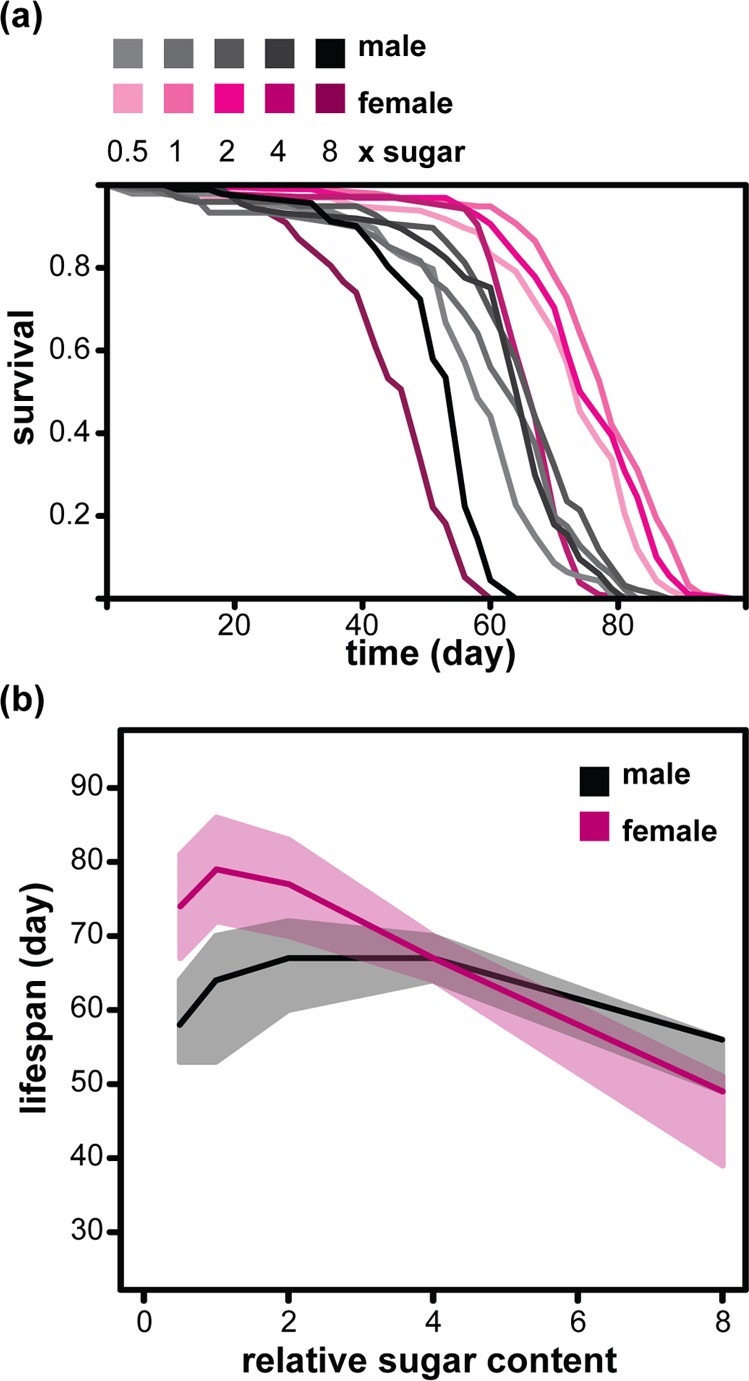
Sexual dimorphism in the response of lifespan to dietary sugar in *Drosophila* (**a**) Survival curves of females and males fed diets with different relative amounts of sucrose (0.5x to 8xS). (**b**) The median lifespans (solid lines) of the same flies with first and third quartiles indicated (shaded area). For statistical analysis see Table 1.

We examined the effects of modulating dietary sugar in males and females of an outbred, healthy, wild-type *Drosophila* population under culture conditions that maximize longevity. Our base-line food contained 10% yeast and 5% sugar [weight/volume; refer to as 1x sugar (1xS), [12]]. The flies were reared at standardized larval density and adults housed in single sex groups, on solid food, 10 individuals per vial. We varied the sugar concentration in the adult diet from 0.5x to 8x the amount present in 1xS. Changing the sugar content of food away from this optimum had a substantial effect on female survival, with 8xS reducing the median lifespan by nearly 40% relative to 1xS (from 79 days on 1xS to 49 day on 8xS, Figure 1a and b), confirming the previous findings [14, 17]. In general, males were shorter lived, with median lifespan of 64 days on 1xS.

Interestingly, their response to dietary sugar was different. The optimum amount of sugar for lifespan was higher (2-4xS, medians of 67 days) and increasing sugar had a substantially smaller effect on males, so that their median lifespan on 8xS was actually higher than the females’ (56 days, Figure 1a and b). Cox Proportional Hazards (CPH) analysis confirmed the significant difference in the way male and female's lifespan responds to dietary sugar (Table 1). Hence, lifespan responds in a sexually dimorphic way to excessive dietary sugar in *Drosophila*.

**Table 1:** Statistical analysis of the data presented in Figure 1a CPH model with 908 dead and 65 censored events.

coefficient	estimate	s.e.	z	p-value
sex (male)	1.56	0.12	13.39	<2×10^−16^
relative sugar (x)	0.48	0.024	19.96	<2×10^−16^
sex : relative sugar	−0.29	0.029	−10	<2×10^−16^

Females were the reference sex, relative sugar was modelled as a continuous variable, “:” indicates the interaction term. The coefficient estimate is the natural log of the hazard ratio where a negative value indicates a beneficial effect on survival.

### Dietary sugar elicits a sexually dimorphic change in feeding behaviour

**Figure 2:**
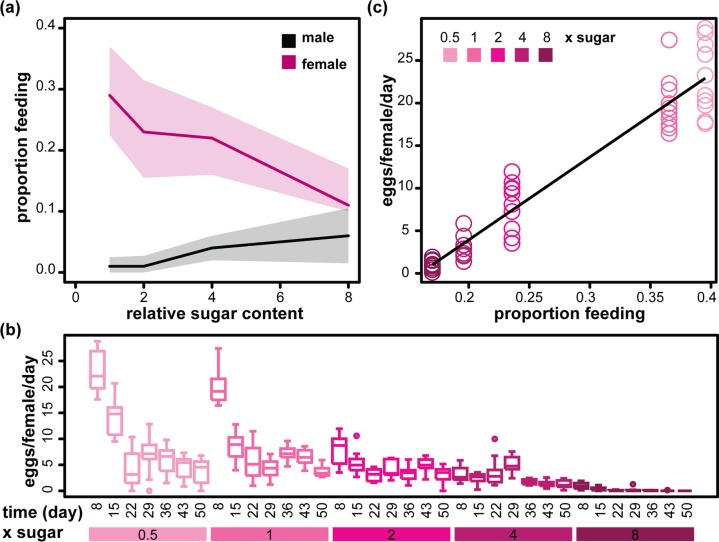
Sexually dimorphic feeding response to sugar and its relationship to female egg laying (**a**) The proportion of male and female flies observed feeding on day 7 on diets with different relative amounts of sucrose (1x to 8xS). Medians (solid line) and first and third quartiles (shaded areas) are shown. Statistical analysis is given in Table 2. (**b**) Bar charts showing the number of eggs laid per female per day on days 8 to 50 (measured every week, n=10) on diets with different relative amounts of sucrose (0.5x to 8xS). (**c**) Egg laying on day 8 (n=10) is correlated to average proportion of flies feeding on day 7. The black line shows the parameters of a LM fit (adjusted R^2^=0.91, p<2×10^−16^).

Flies alter their feeding behaviour in response to the diet presented. Females feed more on diets with higher protein (yeast) and lower sugar content, while males feed more on sugary diets. This is thought to reflect their respective reproductive needs [26]. We also observed that the feeding behaviour on day seven of adulthood was modulated by increasing sugar content (from 1x to 8xS) in a sex-specific way: females decreased their feeding rate in response to sugar, while males increased it, such that while females fed substantially more on 1xS, the difference between males and females was reduced on 8xS (Figure 2a, Table 2). As previously observed [27], males had an overall lower feeding rate (Figure 2a, Table 2), which may be protecting them from the detrimental effects of the diet. However, the feeding rates did not appear to fully account for the sex differences in lifespan. For example, median lifespans were essentially identical between males and females at 4xS (Figure 1b), while feeding rates were different (Figure 2a); lifespan decreased between 4xS and 8xS for both males and females (Figure 1b) while feeding rate increased in males and decreased in females (Figure 2a). However, it remains possible that a part of the sexually dimorphic effect of dietary sucrose on lifespan is mediated by differences in feeding behaviour between males and females.

**Table 2:** Statistical analysis of the data presented in Figure 2a GLM with quasibinomial distribution on 80 observations.

coefficient	estimate	s.e.	t	p-value
intercept	−0.8	0.12	−6.39	1.2×10^−8^
sex (male)	−3.37	0.39	−8.65	6.3×10^−13^
relative sugar (x)	−0.12	0.031	−4.06	1.2×10^−4^
sex : relative sugar	0.31	0.31	4.43	3.1×10^−5^

Females were the reference sex, relative sugar was modelled as a continuous variable (1 to 8xS), “:” indicates the interaction term. The coefficient estimates are logs of odds of feeding, where a negative value indicates a reduction in feeding.

Excessive dietary sugar has been shown to negatively affect female fecundity [12]. Our observations confirmed these findings, revealing that fecundity is particularly susceptible at its peak in early life (Figure 2b). Interestingly, we found a strong linear relationship between feeding rate on day seven and eggs laid on day eight (Figure 2c). Reduced feeding would result in an effective decrease in protein intake and protein is strongly liked to egg production [10, 13, 14, 26, 28]. Hence, our data suggest that a reduction in feeding may mediate a part of the negative effects of high sugar on fecundity, in our experimental paradigm. Overall, our data confirm that dietary sugar elicits sexually dimorphic feeding responses, and indicate that these may have secondary consequences.

### Dietary sugar promotes acquisition of starvation resistance with sexually dimorphic dynamics

**Figure 3:**
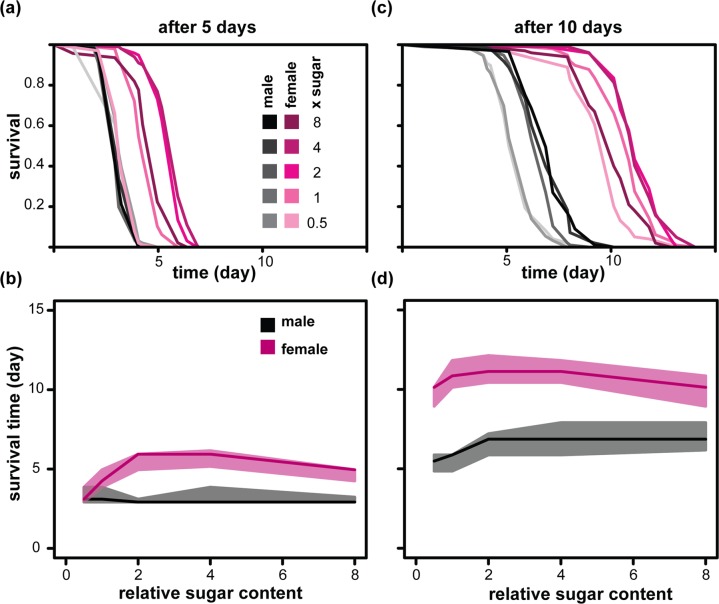
Sexual dimorphism in the acquisition of starvation resistance in response to dietary sugar Starvation resistance of females and males was measured after feeding on the indicated diets for either 5 days [(**a**) and (**b**)] or 10 days [(**c**) and (**d**)]. (**a**) and (**c**) show the survival during starvation with the data summarised in (**b**) and (**d**) where medians (solid lines), first and third quartiles (shaded area) are indicated. Colour codes for (**c**) and (**d**) are shown in (**a**) and (**b**). Statistical analysis is presented in Table 3.

Increase in dietary sugar has been shown to result in increased triacylglycerol (TAG) in females and males [14, 24]. TAG acts as an energy store and could promote starvation resistance. To test the effect of diet on starvation, we fed males and females diets containing 0.5x to 8x sugar for 5 or 10 days before assaying their starvation resistance. We found that in females, 5 days of feeding with high dietary sugar promoted starvation resistance (Figure 3a and b), as previously observed [29]. After 10 days, all females gained a much higher resistance to starvation and the apparent benefits of sugar-rich diets were reduced (Figure 3c and d). On the other hand, male starvation resistance was less influenced by relative dietary sugar after 5 days of feeding than after 10 days of feeding (Figure 3a to c). Overall, increased sugar consumption drove acquisition of starvation resistance with sexually dimorphic dynamics, a conclusion that was confirmed with CPH analysis (Table 3). Hence, the effect observed for lifespan was reversed for starvation resistance but the sexual dimorphism remained: relative to males, the females showed an increased benefit of high dietary sugar for starvation resistance in early life and a greater cost of the same diet for survival in later life.

**Table 3:** Statistical analysis of data presented in Figure 3 CPH model with 1956 dead and no censored events.

coefficient	estimate	s.e.	z	p-value
sex (male)	−0.66	0.29	−2.3	0.023
relative sugar (x)	−0.24	0.05	−4.7	2.4×10^−6^
time on diet (days)	−1.1	0.035	−30.65	<2×10^−16^
sex : relative sugar	0.52	0.063	8.13	4.4×10^−16^
sex : time on diet	0.48	0.04	12.11	<2×10^−16^
relative sugar : time on diet	0.025	0.006	4.14	3.5×10^−5^
sex : relative sugar : time on diet	−0.072	0.008	−9.04	<2×10^−16^

Females were the reference sex, relative sugar and time on diet (both before the start of starvation) were modelled as continuous variables, “:” indicates the interaction term. The coefficient estimate is the natural log of the hazard ratio where a negative value indicates a beneficial effect on survival.

## DISCUSSION

Metabolic differences between the sexes are well documented. Indeed, a recent study found over 15% of metabolites are present in different quantities between males and females in *Drosophila*, and the metabolome as a whole is highly predictive of sex [9]. A part of this is likely to be the direct result of the reproductive investment, where the requirement for a large production of biomass drives females into a more anabolic state. Indeed, males and females in *Drosophila* have different dietary optima for reproduction [26]. In particular, males not only have a higher tolerance of excess carbohydrate in terms of lifespan (this study), but also require higher amount of carbohydrate for optimal offspring production than females [26]. Indeed, several different traits required for reproductive success in males are maximised by a relatively high carbohydrate intake [30]. The sex differences in nutritional optima for reproduction would be expected to result in a sexual conflict, that may have indirect consequences past reproduction. Interestingly, the optimal diet for male reproduction may be quite close to their lifespan optimum, whereas this does not appear to be the case for females. Overall, the distinct nutritional/metabolic demand imposed to maximise reproduction may underlie the differences in response(s) to nutrition between males and females, leaving females’ lifespan more susceptible to the effects of diet, and introducing a gap between the diets that are optimal for female reproduction and lifespan.

At the same time, our data indicate that sugar-rich diets promote starvation resistance in early life at the expense of lifespan in females. It is possible that such a response has evolved to allow the females to survive brief food shortages during their reproductive period. This is similar to the previously proposed idea that the human propensity for obesity is driven by past selection of “thrifty” genotypes that were better able to sustain periods of starvation. Indeed, it has been proposed that obesity in females, whose sites of fat deposition are different to those of males, reflects an exaggeration of adaptations promoting female reproductive success [31]. With respect to flies, however, it is also possible that the increased starvation resistance is a side-effect of having to process a substantial amount of excess sugar present in the diet in order to consume a sufficient amount of protein for reproduction, as proposed under the protein leverage hypothesis [32].

Sugar-rich diets in flies have increasingly been used as a model of unhealthy, contemporary, human diets. While the links between contemporary human diets and a range of negative health outcomes are only coming to light [5], sugar-rich diets in flies have successfully modelled several features of the metabolic syndrome and the associated pathologies, allowing rapid access to the molecular mechanisms of disease [14, 17–24]. Our study adds a new dimension to this research effort by demonstrating that excess sugar in the diet has a sexually dimorphic effect on several traits in adult *Drosophila*. Understanding the mechanistic basis of this dimorphism, in flies and other animals, may help address the causes and consequences of the metabolic syndrome in the human population as a whole.

## MATERIALS AND METHODS

### Fly stocks and husbandry

We used the Dahomey stock, which was collected in 1970 in Dahomey (now Benin) and has been kept in population cages with overlapping generations maintaining its lifespan and fecundity at levels similar to freshly caught stocks. *w^1118^* mutation was back-crossed into the stock. Experimental flies were reared at standardised larval densities on SYA (called here 1xS) which contained 10% yeast, 5% sucrose and 1.5% agar (weight/volume; with preservatives; [12]). All flies were kept and experiments conducted at 25°C, 60% humidity and 12h:12h light:dark cycles. Experimental flies were allowed to mate for 48 h and sorted into single-sex groups in vials containing the indicated food. The food conditions were: 0.5xS (2.5% sucrose), 1xS (5% sucrose), 2xS (10% sucrose), 4xS (20% sucrose) and 8xS (40% sucrose); each containing the same amount of other components as 1xS.

### Lifespan, feeding, egg counts and starvation assays

For lifespan assays, flies were housed at 10 flies per vial, food changed and dead/censored flies recorded 2-3 times a week. Feeding rate was assessed with the proboscis-extension assay during the first 2-3h of light on 7-day old flies (5 days after start of treatment) housed 5 per vial, as described [27]. Eggs were counted in vials that housed 10 females after ∼24h of egg laying and are expressed as eggs laid per female per day. To assess starvation resistance, flies (10 per vial) were transferred onto medium containing 1.5% agar alone after 5 or 10 days of feeding on the indicated diet and the number of dead flies counted 1-2 times a day.

### Statistical analyses

Data were analysed in R (R Core Team [2017]. R: A language and environment for statistical computing. R Foundation for Statistical Computing, Vienna, Austria. URL https://www.R-project.org/). Cox Proportional Hazards (CPH) models were fitted using the *survival* package (http://CRAN.R-project.org/package=survival). Feeding data were analysed with a Generalised Linear Model (GLM) and a quasibinomial distribution. Eggs laid per female per day on day 8 were correlated to average proportion feeding on day 7 with a Linear Model (LM). Details of the models fitted are given in Table captions.
